# Genome-Wide Binding and Transcriptome Analysis of Human Farnesoid X Receptor in Primary Human Hepatocytes

**DOI:** 10.1371/journal.pone.0105930

**Published:** 2014-09-08

**Authors:** Le Zhan, Hui-Xin Liu, Yaping Fang, Bo Kong, Yuqi He, Xiao-bo Zhong, Jianwen Fang, Yu-Jui Yvonne Wan, Grace L. Guo

**Affiliations:** 1 Department of Pharmacology and Toxicology, School of Pharmacy, Rutgers University, Piscataway, New Jersey, United States of America; 2 Department of Pharmacology, Toxicology, and Therapeutics, University of Kansas Medical Center, Kansas City, Kansas, United States of America; 3 Department of Medical Pathology and Laboratory Medicine, University of California, Davis Health Systems, Sacramento, California, United States of America; 4 College of Science, Institute for Computer Applications, Huazhong Agricultural University, Wuhan, Hubei, China; 5 Department of Pharmaceutical Sciences, School of Pharmacy, University of Connecticut, Storrs, Connecticut, United States of America; 6 Biometric Research Branch, National Cancer Institute, Rockville, Maryland, United States of America; IRCCS Istituto Oncologico Giovanni Paolo II, Italy

## Abstract

**Background & Aims:**

Farnesoid X receptor (FXR, *NR1H4*) is a ligand-activated transcription factor, belonging to the nuclear receptor superfamily. FXR is highly expressed in the liver and is essential in regulating bile acid homeostasis. FXR deficiency is implicated in numerous liver diseases and mice with modulation of FXR have been used as animal models to study liver physiology and pathology. We have reported genome-wide binding of FXR in mice by chromatin immunoprecipitation - deep sequencing (ChIP-seq), with results indicating that FXR may be involved in regulating diverse pathways in liver. However, limited information exists for the functions of human FXR and the suitability of using murine models to study human FXR functions.

**Methods:**

In the current study, we performed ChIP-seq in primary human hepatocytes (PHHs) treated with a synthetic FXR agonist, GW4064 or DMSO control. In parallel, RNA deep sequencing (RNA-seq) and RNA microarray were performed for GW4064 or control treated PHHs and wild type mouse livers, respectively.

**Results:**

ChIP-seq showed similar profiles of genome-wide FXR binding in humans and mice in terms of motif analysis and pathway prediction. However, RNA-seq and microarray showed more different transcriptome profiles between PHHs and mouse livers upon GW4064 treatment.

**Conclusions:**

In summary, we have established genome-wide human FXR binding and transcriptome profiles. These results will aid in determining the human FXR functions, as well as judging to what level the mouse models could be used to study human FXR functions.

## Introduction

Farnesoid X receptor (FXR, *NR1H4*) is a ligand activated transcription factor belonging to the nuclear receptor (NR) superfamily [1], and is highly expressed in the liver, intestine, and kidney, both in humans and rodents [2]. Bile acids (BAs) are the endogenous ligands of FXR [3]. FXR mainly functions as the BA sensor by regulating genes that are critically involved in BA homeostasis, including BA biosynthesis, conjugation, and enterohepatic circulation [4]. In addition, it has been shown that FXR is also involved in lipid and glucose homeostasis, inflammation, and tumorigenesis [4]–[7]. FXR normally forms a heterodimer with retinoid X receptor alpha (RXRα) and binds to DNA elements as FXR response elements (FXRREs) [1]. The most common DNA motif bound by FXR is an inverted repeat separated by one nucleotide (IR1). Upon ligand activation, the heterodimer normally activates the expression of its target genes.

Chromatin immunoprecipitation - deep sequencing (ChIP-seq) analysis has been widely used to study the functions of various NRs, including androgen receptor (AR), estrogen receptor alpha (ERα), glucocorticoid receptor (GR) etc. [8]–[10]. This approach has aided in discovering novel pathways regulated by these NRs.

We and others have reported the genome-wide binding analysis of FXR in mice [11]–[13]. These studies suggest broad functions of mouse FXR as well as novel molecular mechanisms, by which FXR regulates its target genes. 1^st^, FXR could bind to multiple sites within a known FXR target gene. For example, FXR binds to both the promoter and 3′ gene regulatory regions of the *Nr0b2* gene, which encodes small heterodimer partner (SHP) [11], and this pattern of binding likely enhances chromatin interaction and subsequent gene expression [14]. 2^nd^, many new target genes of FXR are identified in the liver and/or intestine, including the *Sqstm1* gene, which encodes the protein p62, an important component of autophagy [15]. 3^rd^, FXR cooperates with other transcription factors, most likely orphan nuclear receptors, to modulate transcription of genes involved in specific biological processes. For exp., FXR and LRH-1 (liver receptor homolog-1) co-regulate genes involved in lipid homeostasis [16], [17]. 4^th^, FXR elicits tissue-specific binding patterns, indicating differential regulation of chromatin structures as well as FXR functions among different organs/cells. 5^th^, FXR binding could suppress gene expression, which could be altered during disease state, such as obesity [13]. Taken together, these studies suggest that FXR may regulate diverse physiological and pathological processes in mice, underlying that tissue- or even pathway-specific modulations of FXR may provide better treatment strategies to various lipid- and BA-associated diseases. Indeed, recent literatures have highlighted FXR as a potential therapeutic target for different metabolic diseases, such as parenteral nutrition associated cholestasis [18], vertical sleeve gastrectomy [19], and more commonly nonalcoholic steatohepatitis (NASH) [20], [21], while only limited treatment options are currently available for these diseases.

To date, the binding of human FXR in primary human hepatocytes (PHHs) or hepatoma cell lines has been characterized to limited genes, including *ABCB4* (ATP-binding cassette, sub-family B, member 4), *ABCB11, FGF19* (fibroblast growth factor 19), *ICAM1* (intercellular adhesion molecule 1), *and NR0B2* [4], [22]–[25]. However, the genome-wide FXR binding profile in humans is not yet available. More importantly, little information is known about species similarities and differences in terms of FXR binding between humans and mice, which are needed urgently to determine to what degree the murine models can be used to study the role of FXR in various physiological and/or pathological conditions.

In this study, using ChIP-seq and RNA-seq techniques, we determined the genome-wide binding and transcriptome profiling of human FXR in PHHs. We compared and contrasted the binding patterns and gene regulation profiles of FXR between human and mouse livers.

## Materials and Methods

### Cells and Treatment

Primary human hepatocytes (PHHs) used in this study were obtained through the Liver Tissue Cell Distribution System from the University of Pittsburgh [26], [27]. Only diagnostic and demographic information were obtained and provided by the supplier, no identifier was obtained. Comprehensive information of the PHH donors received in this study was listed in **Table S1.** PHHs were cultured in 37°C, 5% CO_2_ upon arrival. Three hours later, medium were refreshed with serum-free HMM Hepatocyte Maintenance Medium supplemented with dexamethasone, insulin, and GA-1000 (Lonza, Switzerland). Next morning, cells were treated with 5 µM GW4064 [11], a synthetic FXR agonist, or control vehicle, DMSO. Cells were collected for chromatin and RNA isolation at 1 or 24 hours after the treatment, respectively. RNA was isolated using TRI Reagent (Invitrogen, CA), according to the manufacture's instruction. FXR activation was confirmed by the induction of known human FXR target genes using Reverse Transcriptase (RT) quantitative PCR (RT-qPCR). Primer sequences were listed in **Table S2**.

### Chromatin Immunoprecipitation

One hour after GW4064 treatment, cells were fixed in 1% formaldehyde for 10 minutes, followed by quenching with glycine and rinsing with cold PBS. Afterwards, cells were collected and lysed. Nuclei were released and sonicated into 200–700 base-pair (bp) DNA fragments. Aliquot chromatin was incubated overnight with 5 µg anti-FXR antibody (1∶1 mixture of sc-1204x and sc-13063x, ChIP grade) (Santa Cruz Biotechnology, CA), or control rabbit immunoglobulin G (rIgG, sc-2027) (Santa Cruz Biotechnology, CA). Chromatin-antibody complex were pulled down with prewashed Dyna beads (Invitrogen, CA), washed and eluted. DNA fragments associated with FXR or control antibodies were eluted and purified. Input genomic DNA was obtained through similar elution and purification procedures. Quality of ChIP assay was confirmed by qPCR with primers amplifying known FXRREs of human FXR target genes (promoter regions of *BSEP* (bile salt export pump) and *OST-β* (organic solute transporter beta) as well as the negative control (promoter region of *IL-8* (interleukin-8). Primer sequences were listed in **Table S2**. PHH samples from four donors, with good FXR activation and pull-down efficiency, were selected to pool together for the generation of sequencing libraries (**Table S1**).

### Sequencing Library Preparation

Equal amounts of chromatin from the selected four PHH donors were pooled together, followed by ChIP assay to generate DNA for ChIP-seq library preparation. Equal amounts of RNA from the selected PHHs were pooled together as well for RNA-seq library preparation. DNA and RNA sequencing libraries were prepared using the Illumina TrueSeq™ DNA and RNA Sample Prep Kit (Illumina, CA), respectively. The quality of all library samples was confirmed by Agilent Bioanalyzer (Agilent Technologics, CA) before the sequencing reactions. For ChIP-seq, purified library DNA ranging from 400 to 500 bp was fractionated on an agarose gel, followed by extraction and purification before sequencing. All libraries were sequenced 100 bp paired-end on Illumina HiSeq2000 sequencing system.

### ChIP-seq Data Analysis

Genome Analyzer Pipeline Software (Illumina, CA) were used for both primary image data files processing and base calling. All sequenced paired-end reads were aligned to *Homo sapiens* version 19 (hg19) reference genome using bowtie (version 0.12.7) [28]. Only uniquely mapped reads were included. Regions with read enrichment were detected using Model-based Analysis of ChIP-Seq (MACS v 1.4.1) method [29]. By comparing with the rIgG background, non-specific peaks with false discovery rate (FDR) greater than 0.1 were eliminated. Identified peaks were further split by Mali Salmon's Peak Splitter (http://www.ebi.ac.uk/bertone/software.html) and filtered by *p*-value of Poisson distribution lower than 10^−5^. Peaks were annotated using R packages (http://www.r-project.org) based on the ENSEMBL version 65 human genes.

### Motif Analysis for ChIP-seq

For each ChIP-seq dataset, the sequences for the summit regions (201 bp), spanning 100 bp up and downstream from the summit of each peak, were retrieved. The top 500 sequences with the highest peak score were selected for motif analysis based on MEME (Multiple Em for Motif Elicitation) [30].

### ChIP-seq Validation

To validate the ChIP-seq results, we first identified the IR-1 site for each peak, which was located inside the peak summit regions (201 bps) used for motif analysis. Then the genome sequences spanning the IR-1 sites were retrieved to design qPCR primers. ChIPed DNA from individual donors and pooled samples were used for qPCR validation. The primer sequences were listed in **Table S2**.

### Microarray

C57BL/6J (hereafter referred to as wild type (WT)) mice (n = 3) were treated with either GW4064 (100 mg per kg body weight) or vehicle three times as previously described [31] (first dose at 8 am, second dose at 6 pm, third dose at 8 am the second day). Mice were fasted overnight starting from the second dose and liver tissues were collected at 2 hours after the third dose. All mice used for microarray study were maintained in pathogen-free animal facilities in the Laboratory of Animal Research, under a standard 12-hour light/dark cycle (6:00AM/6:00PM) with free access to standard chow and autoclaved tap water. Animal protocols and procedures were approved by the Institutional Animal Care and Use Committee (IACUC) at the University of Kansas Medical Center. Total RNA from livers was prepared with TRIzol Reagent (Invitrogen, CA), and the whole transcription expression levels were determined using Mouse Gene 1.0 ST Array system manufactured by Affymetrix, Inc.. Microarray data were analyzed using the Affymetrix Power Tools (http://www.affymetrix.com).

### RNA-seq Data Analysis

For RNA-seq in PHHs, RNA was pooled from selected PHH donors. After sequencing, the obtained reads were aligned to the *Homo Sapiens* reference genome (hg19) using TopHat (version 2.0.0) [32]. The resulted alignments were then assembled into transcripts using Cufflinks (version 2.0.2). Cuffdiff, a component of the Cufflinks package, was used to estimate FPKM (fragments per kilobase of exon model per million mapped fragments) and identify differentially expressed transcripts. Finally the Baggerley's test was used to perform the differential expression analysis.

### Pathway Analysis for ChIP-seq and RNA-seq

Functional genes from ChIP-seq and RNA-seq were selected and analyzed using the Functional Annotation Tool in DAVID ((http://www.david.niaid.nih.gov). For a pathway or process to be defined, the threshold count was set at 2 with a minimum EASE (Expression Analysis Systematic Explorer) score, a modified Fisher Exact Test, of 0.1. Categories from DAVID with false discovery rates (FDRs) less than or equal to 0.1 were considered as statistically significant.

### Data Files Access

All sequencing data files discussed in this publication have been deposited in NCBI's Gene Expression Omnibus [33] and are accessible through GEO Series accession number GSE57312 (http://www.ncbi.nlm.nih.gov/geo/query/acc.cgi?acc=GSE57312)

### Statistical Statement

For RT-qRCR experiments, due to the difficulty of repeating sample collection from individual PHH donors, PHHs from different donors served as experimental replicates to validate FXR activation for the pooled PHH samples.

## Results

### FXR Activation in PHHs

To identify genome-wide FXR binding sites in primary human hepatocytes, we first validated FXR activation in the PHHs obtained in this study. Upon 24 hours of GW4064 treatment, mRNA levels of classic FXR target genes (BSEP, OST-β) were induced in PHHs from individual donors (**Figure S1A**). Pooled chromatin samples, which were collected after 1 hour GW4064 treatment, showed significant enrichment of FXR binding to known FXR targets (promoter regions of *BSEP* and *OST-β*), but not the negative control (promoter region of *IL-8*) (**Figure S1B**). ChIPed-DNA, generated from pooled chromatin from selected donors (**Table S1**), as well as pooled RNA was then used to generate DNA and RNA sequencing libraries. Indeed, many known human FXRREs were detected with relatively high peak values in this study, in both DMSO and GW4064 treated PHHs (**Table 1**). Again, multiple FXR binding sites were found in the *NR0B2* and *OST-β* gene in our datasets (**Table 1**), which resembled the binding patterns of FXR to these genes in mice [11].

**Table 1:** Selected previously reported FXR target genes identified in this study*.

	PHH-DMSO		PHH-GW	
Gene	Distance To TSS	Binding Score	Distance To TSS	Binding Score
*ABCB11*	−83	69	−103	319
*FGF19*	3665	89	3695	251
*ICAM1*	58	91	88	111
*MIR122*	−36936	89	−36941	294
*NR0B2*	−258	83	−178	112
	3252	41	3212	69
*OST*−*β*	−48	67	17	68
	10087	67	10017	138
*PPARα*	−2839	126	−2789	203

* Distance To TSS is the distance of the peak of the binding site to the transcription start site (TSS) of the corresponding RefSeq gene. Note that the peak identified from ChIP-seq analysis may not overlap exactly with the IR-1 motif found from motif analysis. The binding score is the FXR antibody pull-down score normalized to rIgG control antibody generated by the sequencing analysis processes. Note that for most FXR targets listed, the binding score retrieved from PHH-GW dataset is much larger than the score from PHH-DMSO for the same peak. This is a general trend for most shared FXR targets between the two datasets. Genes with their full names: *MIR122* (microRNA 122), *PPARα* (peroxisome proliferator-activated receptor alpha).

In ChIP-seq, the peak summit of each peak (binding site) was a single bp position within the peak with the highest coverage given by the MACS analysis. Due to the relatively large size of fragmented library DNA obtained in this study (average 350 bp for ChIP-seq), and the subtle differences in the local chromatin environment in different samples, we saw slightly different peak width and peak summit values for the same binding sites in the two datasets. For example, for the binding site located in the intron of *FGF19* gene, the peak width was 1000 and 851 bps in the ChIP-seq datasets from DMSO treated PHHs (PHH-DMSO) and GW4064 treated PHHs (PHH-GW), respectively. And the peak summits in the two datasets were 3665 and 3695 bps downstream from the TSS of *FGF19*, respectively. Nevertheless, these peaks were indicating the same binding site.

### Comparison of Global FXR Binding between PHHs and Mouse Livers

**Figure 1:**
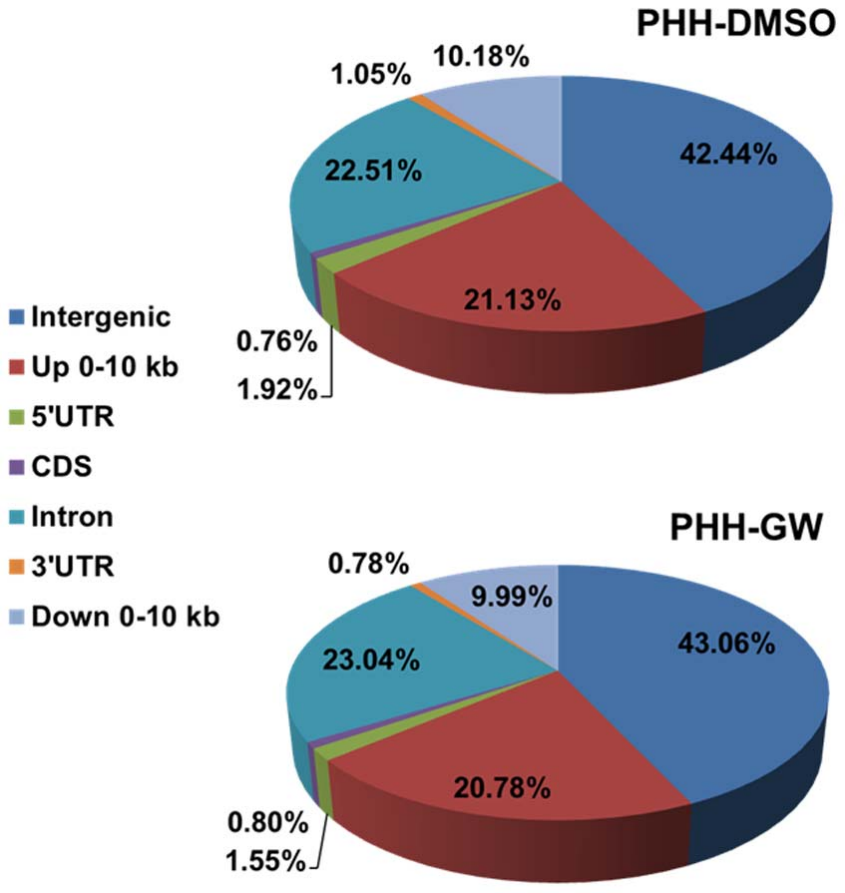
Genomic distribution of FXR binding sites in PHH-DMSO and PHH-GW. Percentage of FXR binding sites in the two datasets that were distributed to >10 kb from genes (intergenic), 0–10 kb upstream of genes (Up 0–10 kb), 5′UTRs, coding sequence (CDS), introns, 3′UTRs, and 0–10 kb downstream of genes (Down 0–10 kb) were shown. The cut off score for the data analysis presented in **Figure 1** **,** **2** **,** **3** and **5** were 20 from ChIP-seq data analysis.

**Figure 2:**
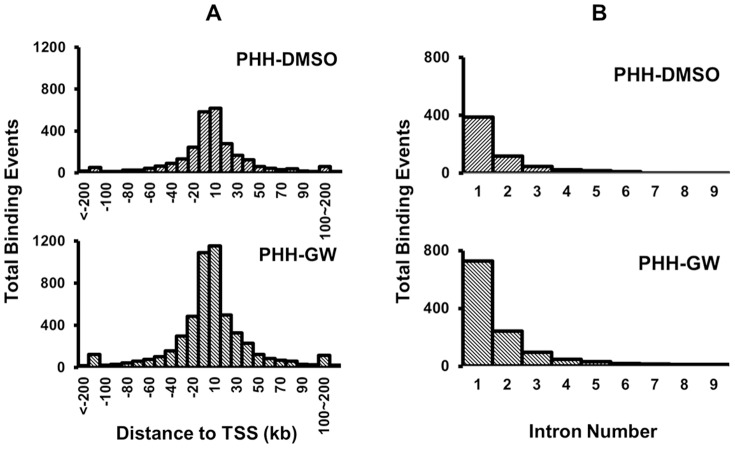
Distribution of total FXR binding sites relative to TSSs, and intron binding profiles of FXR in the two datasets. (A) The left panel shows the frequency distribution of FXR binding. The number of binding events (y-axis) was plotted against the distance from TSSs in 10 kb increments (x-axis) for PHH-DMSO and PHH-GW. (B) The cumulative binding events of FXR distributed only to introns of RefSeq genes in the two datasets. The graph displays the total number of FXR binding peaks (y-axis) in PHH-DMSO and PHH-GW located within intron 1-9 of RefSeq genes (x-axis). Total of 62.4% and 60.2% of intron binding events were located in the first introns in PHH-DMSO and PHH-GW, respectively.

When cut off score (CO score) for ChIP-seq data analysis was set as 20, a total of 2759 and 5235 FXR binding sites were identified from PHH-DMSO and PHH-GW, respectively. Human and mouse FXR binding profiles in livers were compared between these human data with our previous genome-wide mouse FXR binding data, which were obtained from WT mice treated with GW4064 (referred to as mLiver-GW, which was not normalized to the rIgG control though) [11]. The following results were obtained by comparing the global binding pattern in these datasets: 1^st^, genomic distributions of FXR binding sites were similar in PHHs compared to those in mice (**Figure 1**). Briefly, around 43% peaks were located in intergenic, 21% in upstream 0-10 kb, 22% in introns, 10% in downstream 0–10 kb, 2% in 5′ untranslated region (UTR), 1% in 3′ UTR, and 1% in coding DNA sequence (CDS) regions of their associated RefSeq genes in both PHH-DMSO and PHH-GW. This site-distribution pattern was similar to that in mice. Though in mouse livers, around 30% peaks were located in introns and 15% in upstream 0–10 kb region [11]. 2^nd^, the distribution patterns of total FXR binding sites relative to transcription start sites (TSSs) of the associated RefSeq genes, and FXR's intron binding patterns were both similar to those in mice as well. The highest frequency of total binding events was located within 0–10 kb up and downstream of TSSs (**Figure 2A**). Most intron binding events were located in the 1^st^ intron and the number of total binding events in individual intron decreased as the intron number increased (**Figure 2B**). These patterns were almost identical to those in mice [11].

### Motif Analysis of FXR Binding Sites in PHHs

**Figure 3:**
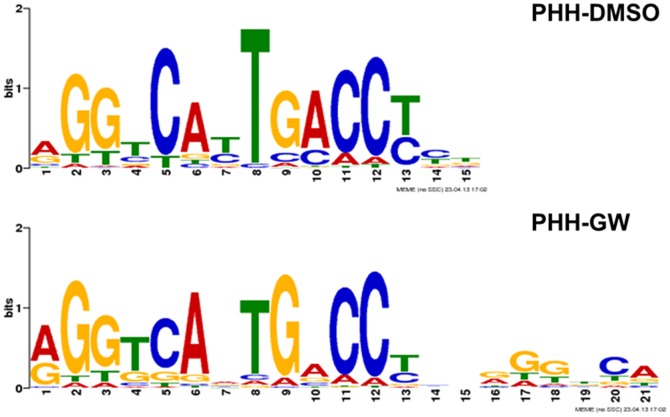
Motif analysis. The most commonly identified sequence motifs from the top 500 FXR binding sites in the two datasets using MEME. These motifs were found in totally 247, 240 sites in PHH-DMSO, PHH-GW from the top 500 peaks (*p*-value <1.00e-5), respectively. It is interesting that there is a putative nuclear half site next to the IR-1 site from PHH-GW, but not in PHH-DMSO.

The most commonly reported FXR binding motif is an IR1 in both mice and humans. This motif has been reported in many human FXR target genes, such as *ABCB11, FGF19, NR0B2,* and *OST-β* [22], [23], [34]. When we select the top 500 peaks from PHH-GW and PHH-DMSO, the most common motif found in PHH-GW was an IR-1 with a putative nuclear half site, whereas in PHH-DMSO it was the IR1 motif (**Figure 3**). And interestingly, when we select the top 501–1000 peaks to run motif analysis, we only obtained the IR-1 motif from both datasets (data not shown). The presence of IR-1 with nuclear half site was also similar to our previous motif analysis in mouse livers [11].

### Validation of ChIP-seq and Novel FXR Targets

After motif analysis, we were able to precisely locate the IR-1 site associated with each peak summit for most peaks in our datasets. The subtle position difference (mostly around 50 bp) from the IR-1 site to the peak summit for each FXR target was most likely caused by a combination of relatively large DNA fragments used for our sequencing analysis and the technical limitation of the sequencing processing and data analysis. Since most classic FXR targets were presented correctly in the datasets (**Table 1**), we then focused on validating novel FXR targets.

**Figure 4:**
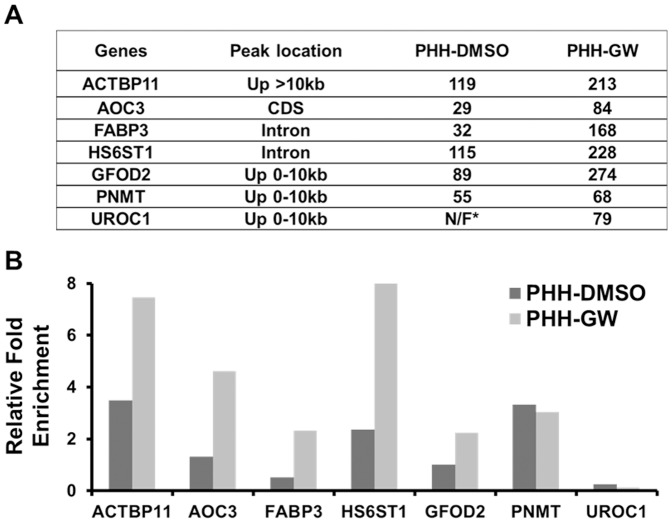
ChIP-seq validation. The location of FXR binding sites (second column on the left) and binding scores (the two columns on the right) for the selected novel FXR targets found in PHH-DMSO and PHH-GW were summarized in (A), and ChIP-qPCR results for these targets from pooled GW4064 or DMSO treated PHHs were presented in (B). FXR pull-down was normalized to rabbit immunoglobulin G control. Note that *ACTBP11* is a pseudogene in humans. GW4064 treatment also induced the mRNA levels of *AOC3*, *FABP3*, *PNMT* and *UROC1* in PHHs in RNA-seq (data not shown). *N/F stands for not found. Genes with their full names: *ACTBP11* (actin, beta pseudogene 11), *AOC3* (amine oxidase, copper containing 3), *FABP3* (fatty acid binding protein 3), *HS6ST1* (heparan sulfate 6-O-sulfotransferase 1), *GFOD2* (glucose-fructose oxidoreductase domain containing 2), *PNMT* (phenylethanolamine N-methyltransferase), and *UROC1* (urocanate hydratase 1).

ChIP-qPCR was performed on chromatin samples from pooled PHH samples, as well as individual PHH donors. For many FXR targets, we were unable to detect valid Ct value from rIgG control from qPCR experiments for individual PHH donors, mainly due to limited quantity of chromatin samples. Nevertheless, we were able to confirm enhanced FXR pull-down from GW4064 treated PHHs comparing to DMSO control, for the promoter regions of *BSEP* and most selected novel targets, but not the negative controls. And for those FXR targets, of which FXR pull-down was not further enhanced upon GW4064 treatment, we were able to calculate their FXR pull-down efficiency after normalizing to rIgG control from pooled PHH samples (**Figure 4A, 4B**). Without valid Ct values from the rIgG control pull-down, we couldn't differentiate these FXR targets from the negative controls. For exp., the binding score for PNMT (phenylethanolamine N-methyltransferase) in PHH-DMSO and PHH-GW were 55 and 68, respectively, which were close. Indeed, ChIP-qPCR showed similar FXR pull-down efficiency from pooled PHH samples (**Figure 4B**, around 3 fold for both control and treatment). The promoter region of *OST-β* showed the same trend as well (**Figure 1B**). In this regard, to better illustrate FXR binding in both DMSO and GW4064 treated PHHs, only ChIP-qPCR data for pooled PHHs were presented. We were able to validate FXR pull-down for most selected novel targets with relatively high binding scores (9 out of 11 peaks were validated, with binding scores equal to or above 50 in either PHH-DMSO or PHH-GW (**Figure 4A**). This trend was consistent with our previous findings in mouse livers [11].

### Microarray, RNA-seq and their Correlation with ChIP-seq Datasets

**Figure 5:**
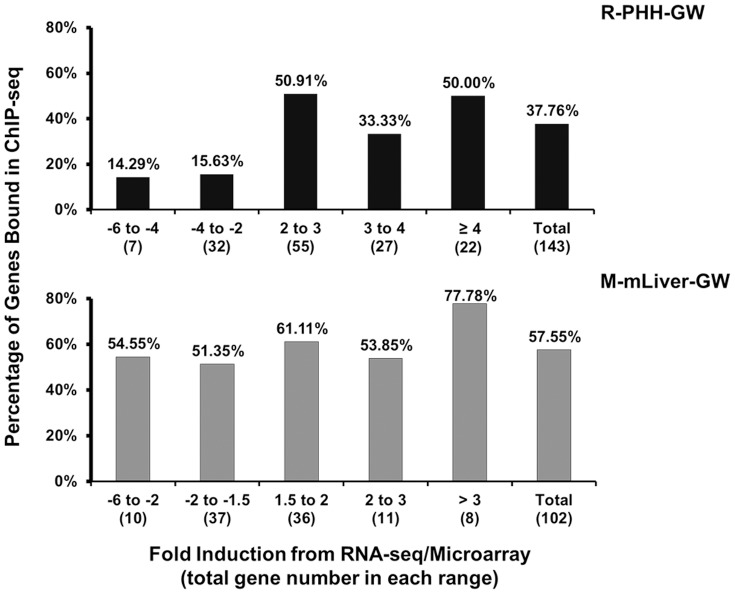
Correlation of FXR binding with target gene expression. The binding of FXR to its target genes were correlated with genes that showed altered mRNA expression levels in RNA-seq for PHHs (R-PHH-GW) and microarray for mouse livers (M-mLiver-GW). The x axis displays the divided range of fold induction in R-PHH-GW or M-mLiver-GW. For RNA-seq, 143 altered genes with log2 fold change ≥2 (fold change ≥4, both up- and down- regulated), *p*-value <0.05 were used, whereas for microarray, 102 altered genes with fold change >1.5 (both up- and down- regulated), *p*-value <0.05 were used. The total number of genes from microarray or RNA-seq analysis in each fold range was listed in parenthesis. The y axis displays the percentage of genes found in M-mLiver-GW and R-PHH-GW, which were also bound by FXR in PHH-GW (top) and mLiver-GW (bottom). Note that for the y axis, the log2 fold change from RNA-seq data analysis was displayed for R-PHH-GW, while for M-mLive-GW, the actual fold change generated from microarray data analysis was displayed.

Using microarray, we obtained a gene expression profile from WT mouse livers treated with GW4064 (M-mLiver-GW) normalized to vehicle control. When set cut off fold induction (CO fold) as 1.5 and *p*-value <0.05 (unpaired *t*-test), we obtained 102 different genes with altered expression levels, up or down more than or equal to 1.5 fold. From RNA-seq for GW4064-treated PHHs (R-PHH-GW), which was normalized to DMSO control, we obtained 143 genes with log_2_ fold enrichment ≥2 (fold change ≥4, both up- and down- regulated) and *p*-value <0.05. The percentage of genes found in microarray and RNA-seq, which were also bound by FXR from ChIP-seq, was plotted in **Figure 5** based on the fold induction from microarray and RNA-seq. Among all the genes found in M-mLiver-GW, over 50% were bound in mLiver-GW for both up- and down- regulated. However in R-PHH-GW, around 50% up-regulated genes were bound in PHH-GW, whereas only a few down-regulated genes were actually bound in PHH-GW. Interestingly, *FGF19* and *CYP7A1* (cytochrome P450, family 7, subfamily A, polypeptide 1) were the top up- and down-regulated FXR targets in R-PHH-GW, respectively.

### Pathway Analysis for ChIP-seq and RNA-seq

A major difference between the mLiver-GW dataset and the PHH-GW dataset was that, more than 5,000 genes and 10,000 peaks were found in mLiver-GW compared to 5,231 peaks identified in PHH-GW. The lack of IgG control for the mLiver-GW may well explain this major difference as non-specific peaks may present in the mouse FXR binding study. Another difference was that the majority of the peaks in mLiver-GW were associated with RefSeq genes, which encode proteins with known functions, whereas in PHH-GW only around 50% peaks were.

In order to compare and contrast the functional detail of FXR binding in humans and mice, two major analyses from DAVID were performed, the Kyoto Encyclopedia of Genes and Genomes (KEGG) and the Gene Ontology Biological Process (GO-BP) analysis. Similar to our previous study, functional genes associated with peaks located in the upstream 0-10 kb promoter regions were selected to run DAVID analyses. Overall, more categories were enriched in PHH-GW than PHH-DMSO. And most categories enriched in PHH-DMSO were also presented in PHH-GW. In this regard, only categories and their corresponding FXR targets from PHH-GW were presented and compared with mLiver-GW. All significantly enriched KEGG categories (with FDR <0.1) (**Table 2A**), and the corresponding genes (**Table S3A**) obtained were listed, whereas most non-redundant categories (with gene count >5) from GO-BP were presented (**Table 3B, S** **3B**). From DAVID analyses, we could see overall similar pathways enriched from mLiver-GW and PHH-GW, though the number of genes retrieved from PHH-GW was smaller. Nevertheless, the percentage of genes found in each category was similar between the two datasets.

**Table 2:** Comparison of DAVID Functional Annotation for PHH-GW versus mLiver-GW*.

A. KEGG analysis						
	mLiver-GW (Total 970 genes)			PHH-GW (Total 343 genes)		
Term	Count	%	FDR	Count	%	FDR
Retinol metabolism				12	2.836879433	0.000335516
Drug metabolism	23	1.82E+00	0.000169359	12	2.836879433	0.001458046
Complement and coagulation cascades	21	1.660079051	0.003343182	11	2.600472813	0.029459911
Metabolism of xenobiotics by cytochrome P450	18	1.422924901	0.030917603	10	2.364066194	0.057413681
PPAR signaling pathway	26	2.055335968	4.11311E-06	7	1.654846336	1.58E+01

B. GO-BP analysis						
	mLiver-GW (Total 970 genes)			PHH-GW (Total 343 genes)		
Term	Count	%	FDR	Count	%	FDR
organic ether metabolic process	11	0.869565	3.11774	13	3.073286	0.0000104
triglyceride metabolic process	11	0.869565	0.268143	12	2.836879	0.0000112
glycerolipid metabolic process	21	1.660079	1.408123	19	4.491726	2.53E-04
lipid transport	26	2.06E+00	0.001436	18	4.255319	2.58E-04
chemical homeostasis	45	3.557312	0.750307	31	7.328605	0.029556
monocarboxylic acid transport	8	0.632411	0.387526	9	2.12766	0.057257
response to wounding	53	4.189723	0.000434	30	7.092199	0.138013
oxidation reduction	86	6.798419	0.000218	34	8.037825	0.138493
cholesterol metabolic process	17	1.34E+00	0.045242	11	2.600473	1.88E-01
homeostatic process	70	5.53E+00	0.036206	37	8.747045	2.92E-01
steroid metabolic process	41	3.241107	2.94E-09	16	3.782506	0.337117
cellular amino acid derivative metabolic process	29	2.29249	0.00108	12	2.836879	5.666095
fatty acid metabolic process	41	3.241107	0.000000255	11	2.600473	40.37399
regulation of cellular ketone metabolic process	8	0.632411	1.457816	9	2.12766	0.151048
acute-phase response	14	1.11E+00	0.0000783	9	2.12766	1.02E-02
acute inflammatory response	26	2.06E+00	0.000000314	10	2.364066	1.44E+00
coenzyme metabolic process	30	2.371542	0.000427	9	2.12766	51.62624
organic acid catabolic process	27	2.134387	5.02E-08			
glucose metabolic process	32	2.529644	0.000019			
cellular amino acid catabolic process	19	1.501976	0.000029			
hexose metabolic process	35	2.77E+00	0.0000489			
monosaccharide metabolic process	36	2.85E+00	0.000351			
L-phenylalanine catabolic process	6	0.474308	0.018543			
pyruvate metabolic process	10	0.790514	0.059427			

^*^Binding sites that were associated with 0-10 kb upstream of RefSeq genes were selected for DAVID functional annotation analyses. The cut off score for ChIP-seq datasets was 20. Totally 970 and 343 RefSeq genes were retrieved from mLiver-GW and PHH-GW, respectively. The categories were listed based on the FDR values from DAVID analyses for PHH-GW dataset.

**Table 3:** DAVID functional annotation for PHH RNA-seq*.

A. KEGG analysis		
Term	%	Genes
Retinol metabolism	2.9508	*CYP1A1, CYP26B1, ADH1C, DHRS9, ADH1B, CYP26A1, CYP2A7, CYP1A2, UGT2B10, RDH16*
Cytokine-cytokine receptor interaction	4.918	*CXCL2, CX3CL1, EDAR, CCL15, CCL18, CXCL10, INHBB, CCL25, TNFRSF9, INHBA, TNFSF10, TNFRSF1B, CCL14, CCL20, CXCL13, IL1B*
PPAR signaling pathway	1.9672	*PPARD, HMGCS2, CYP7A1, FABP3, FABP6, ANGPTL4*
Steroid hormone biosynthesis	1.6393	*HSD3B2, CYP1A1, CYP7A1, UGT2B10, SULT1E1*
Tryptophan metabolism	1.3115	*KYNU, CYP1A1, IDO2, CYP1A2*
Drug metabolism	1.3115	*XDH, UPP1, CYP2A7, UGT2B10*
Tyrosine metabolism	1.3115	*PNMT, ADH1C, ADH1B, TAT, AOC3*
Calcium signaling pathway	2.623	*ADRB1, CYSLTR1, PHKA1, CACNA1H, BDKRB2, VDAC1P1, ITPKA, HTR2A*
Chemokine signaling pathway	2.623	*CCL25, CCL14, CCL20, CXCL13, CXCL2, CX3CL1, CCL15, CCL18, CXCL10*

B. GO-BP analysis		
Term	%	Genes
chemotaxis	4.2623	*CYSLTR1, CXCL2, CX3CL1, CCL15, CCL18, CXCL10, CCL25, CCL14, CCL20, CXCL13, IL1B, LECT2, DEFB1, FGF2*
oxidation reduction	7.8689	*HSD3B2, XDH, ALDH8A1, STEAP4, ADH1C, ADH1B, BBOX1, CYP7A1, CYP26B1, HSD17B6, LOXL4, ALDH6A1, CYP1A1, IDO2, DHRS9, CYP26A1, CYP1A2, CYP27C1, DIO3, ALOX15B, HAO2, CYP2A7, RDH16, DCXR, AOC3*
ion homeostasis	5.2459	*KNG1, MCHR1, PPARD, CYSLTR1, ATP1A2, BDKRB2, CCL15, S1PR3, CCL14, RHCG, CXCL13, MT2A, LGI4, IL1B, NPPB, NEDD4L, MT3*
immune response	5.9016	*ICAM1, KYNU, IGHG4, AQP9, CXCL2, CX3CL1, CCL15, CCL18, CXCL10, CCL25, TNFSF10, TNFRSF1B, CCL14, CCL20, CXCL13, SEMA7A, IL1B, DEFB1, CD14*
regulation of programmed cell death	6.5574	*KNG1, NUAK2, EEF1A2, BCL2A1, ACTN2, BDKRB2, SOX9, DAPK2, GDNF, INHBA, SERPINB9, TNFRSF9, TNFSF10, ALOX15B, TNFAIP8, IL1B, CFDP1, FGF2, PHLDA1, ANGPTL4*

*Functional genes with log_2_ fold enrichment ≥1 (fold change ≥2, both up- and down- regulated) and *p*-value <0.05 were selected for DAVID analysis, totally 291 RefSeq genes were retrieved from the PHH RNA-seq dataset (R-PHH-GW).

For R-PHH-GW, 291 functional genes with log_2_ fold ≥1 (fold change ≥2, up- and down- regulated), and *p*-value ≤0.05 were retrieved for KEGG and GO-BP analyses. Note that the cut off fold change used for pathway analysis is smaller than the cut off used for the ChIP-seq/RNA-seq correlation analysis. For pathway analysis, the cumulative effect from many altered genes in a single pathway could be of functional importance as well, though the level of fold change for individual gene was relatively low. All categories from KEGG and non-redundant categories from GO-BP, and the corresponding genes were presented (**Table 3A, 3B**). In agreement with previous correlation study presented in **Figure 5**, both similar and different categories were enriched from R-PHH-GW. Interestingly, many genes involved in chemokine signaling pathway (KEGG) and chemotaxis (GO-BP) were enriched in R-PHH-GW (**Table 3**), and most of these were not directly bound by FXR.

## Discussion

In this study, combining the widely used ChIP-seq and RNA-seq techniques, we have characterized the genome-wide FXR binding and transcriptome profiles upon ligand activation in selected PHHs. We detected almost all previously identified important human FXR targets, which have diverse physiological functions. Comparing the global FXR binding patterns, we showed that the patterns in PHHs were very similar to those identified in mouse livers in terms of genomic distribution, intron binding pattern, and the association with TSSs of RefSeq genes. These phenomena were in agreement with the conserved function of FXR in transcriptional regulation.

Most convincingly, the motifs found in this study were almost identical to those found in mice. Interestingly, the putative nuclear receptor half site was enriched significantly only from the top 500 peaks in PHH-GW, neither in the top 501 to 1000 peaks nor the top 500 peaks from PHH-DMSO (**Figure 3**). In the chromatin level, the co-binding of FXR and other transcription factors to certain targets may potentially correspond to higher levels of pull-down from ChIP assay, leading to higher enrichment scores for these genes. Previous genome-wide binding analysis of LRH-1 in mice has shown that LRH-1 could bind to the nuclear half site next to IR1, and co-regulate transcription of FXR target genes involved in lipid metabolic processes in mice [16]. The top 500 peaks identified in this study are associated with genes not only involved in lipid metabolism, but in diverse cellular processes. This could indicate a common mechanism of how FXR regulate gene transcription in different cellular processes, while with different cofactors involved. This type of co-regulation has been well studied for ERα [35]. Moreover, the presence of a nuclear receptor half site adjacent to the IR-1 in both PHH-GW and mLiver-GW indicates the similarities of FXR functions from mice to humans in a greater extent by indicating the existence of similar type of cofactors for FXR in different species. This mechanistic similarity implicates that tissue- and even pathway- specific FXR modulation in mice can be translated into therapeutic benefits in humans.

Using multiple pathway analysis tools (KEGG and GO-BP from DAVID), the current study predicts that human FXR could participate in the regulation of diverse physiological processes (**Table 2**, **S3**). Furthermore, similar pathways were enriched from PHH-GW compared to mLive-GW. More genes were obtained from mLiver-GW than PHH-GW. As a result, more pathways were enriched in mLiver-GW [11]. Future studies are needed in order to determine to what degree the lack of IgG control contributes to the increased output from mLiver-GW. Overall, the comparison studies presented in this study will be valuable information for researchers in correlating and translating previous and future mouse FXR studies to human FXR functions.

RNA-seq analysis for DMSO and GW4064 treated PHHs also further confirmed the reliability of GW4064 treatment and FXR activation in this study. Interestingly, *FGF19* and *CYP7A1* were the top up-regulated and down-regulated target genes in PHHs, respectively, whereas in mice *Fgf15* is only found to be expressed and induced in the intestine [31].

When correlating the results from ChIP-seq with RNA-seq for PHHs, and microarray for mouse livers, different trends were observed for genes down-regulated following GW4064 treatment (**Figure 5**). In addition, only a small portion of target genes showed similar change in R-PHH-GW and M-mLiver-GW. This difference may be due to several reasons. First, there are different baseline regulatory network in different species, such as the different expression patterns of FGF19 in humans versus Fgf15 in mice. Besides, the genetic background of the PHH donors could be heterogeneous since we didn't receive certain donor information upon tissue collection, such as races, patient condition, etc. And the inbred C57BL6/J mice were relatively homogenous. Second, for many FXR target genes, the expression levels may be already high in control mice due to activation of FXR by the largely stable bile acid pool, while the levels of residual bile acids in PHHs could be very low. In this regard, GW4064 treatment wouldn't further induce the expression of these genes in mice. In line with this, for many FXR target genes in mice, we detected similar FXR binding from ChIP assay when comparing GW4064 treated versus vehicle control treated mouse livers (data not shown). But we did see dramatic increase of FXR binding from GW4064 versus DMSO treated PHHs (see data in **Table 1**, **Figure 4** and **Figure S1**). Indeed, both the magnitude of relative fold change levels and the number of altered genes were much larger in R-PHH-GW than M-mLiver-GW. Third, gene expression in the mouse livers could be affected by whole body physiology, such as circadian rhythms, hormones, fast/feeding cycles, physical activity and energy level, etc. Fourth, for microarray study, we cannot completely rule out the contributions from other cell types in mouse livers, such as endothelial cells and liver Kupffer cells, while in the enriched PHHs, the number of other liver cell types was minimal. Finally, technical differences between RNA-seq and microarray could be another minor factor as well. Among all these factors, the first two that affecting the baseline expression levels of many FXR target genes, could be the major causes of the differences we saw between the *in vivo* and *in vitro* studies. While certain technical limitations existed, the correlation/comparison study still provides valuable information for human FXR function. For R-PHH-GW, mRNA level down-regulation was observed for only a few FXR target genes found in ChIP-seq. But for M-mLiver-GW, the percentage of up- and down- regulated genes also presented in ChIP-seq was similar. Lee et al have shown that direct gene suppression by FXR is common in mice [13]. The correlation study presented here implicates that FXR may play less important roles in direct gene suppression in humans.

The FXR gene sequence is highly conserved across species, and the protein sequence is very similar between humans and mice [36], [37]. This similarity indicates the overall conserved and important functions of FXR in different species, further confirmed by our genome wide binding studies. On the other hand, the differences between species, from genome landscape, cellular components, all the way to physiology and pathology could contribute to the differences identified in this study, especially for gene expression.

In summary, we have obtained valuable information of genome-wide binding and transcriptome analyses of human FXR in PHHs. Detailed analysis of the ChIP-seq data indicates that the global binding patterns of FXR in PHHs are similar to those in mouse livers. In addition, similar biological pathways were enriched from genes bound by FXR in PHHs compared to those enriched in mouse livers. We also identified and validated novel FXR target genes, with and without alteration of mRNA levels. Species differences were found for specific pathways and within gene families involved in similar pathways. In a major extent, mouse model is a suitable model for studying human FXR functions.

## Supporting Information

Figure S1 **Validation of FXR activation in individual PHH donors and pooled PHH chromatin.** (A) Relative mRNA levels of FXR and FXR targets (*BSEP*, *OST-β*) in the selected four PHH donors (1958, 1959, 1962, and 1974) upon 24 hr GW4064 treatment normalized to DMSO control by RT-qPCR analysis. For each PHH donors, we treated 3 wells of cells with GW4064, 3 with DMSO control. RNA from each well was collected and analyzed individually. Human *18S* was used as the normalization control. (B) ChIP-qPCR analysis of FXR antibody pull-down for the promoter regions of *BSEP*, *OST-β* and *IL-8* upon 1 hr DMSO or GW4064 treatment for pooled chromatin from the selected 4 donors in (A). Fold enrichment of FXR binding was normalized to rabbit immunoglobulin-G control antibody.
(TIF)

Table S1 **Summary of PHH Donors.**
(DOCX)

Table S2 **Primers Used for Quantitative PCR.**
(DOCX)

Table S3 **Comparison of Genes from Selected Categories in DAVID Anotation for ChIP-seq.**
(DOCX)
