# Supplementary figures and images for: Genome-Wide Binding and Transcriptome Analysis of Human Farnesoid X Receptor in Primary Human Hepatocytes
Source: PLoS One. 2014 Sep 8;9(9):e105930. doi: 10.1371/journal.pone.0105930 (PMC4157742; doi:10.1371/journal.pone.0105930)

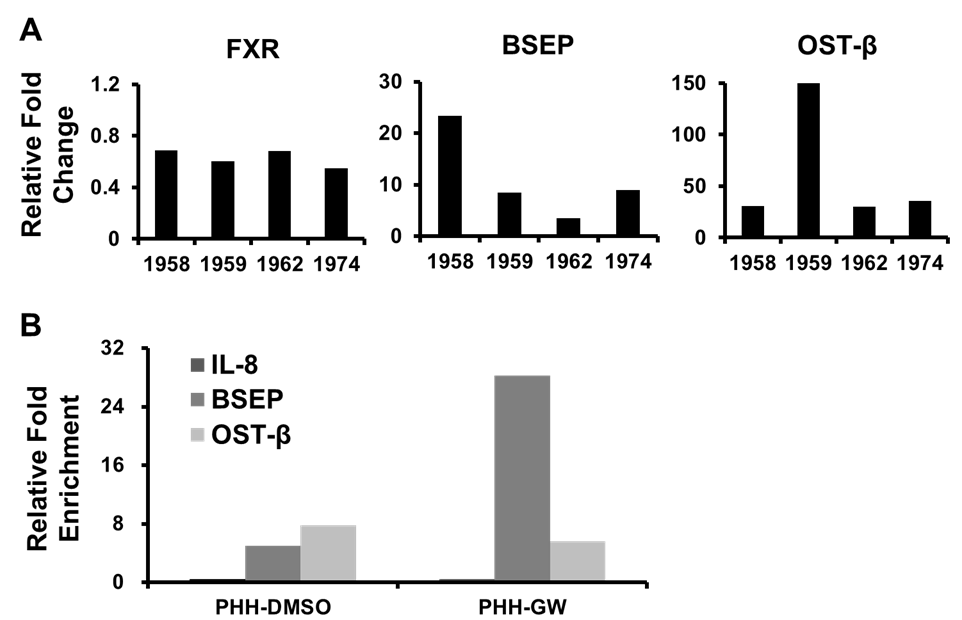

Supplement: Figure S1 — Validation of FXR activation in individual PHH donors and pooled PHH chromatin. (A) Relative mRNA levels of FXR and FXR targets (BSEP, OST-β) in the selected four PHH donors (1958, 1959, 1962, and 1974) upon 24 hr GW4064 treatment normalized to DMSO control by RT-qPCR analysis. For each PHH donors, we treated 3 wells of cells with GW4064, 3 with DMSO control. RNA from each well was collected and analyzed individually. Human 18S was used as the normalization control. (B) ChIP-qPCR analysis of FXR antibody pull-down for the promoter regions of BSEP, OST-β and IL-8 upon 1 hr DMSO or GW4064 treatment for pooled chromatin from the selected 4 donors in (A). Fold enrichment of FXR binding was normalized to rabbit immunoglobulin-G control antibody. (TIF) [file pone.0105930.s001.tif]
