# Supplementary material for: Genome-Wide Binding and Transcriptome Analysis of Human Farnesoid X Receptor in Primary Human Hepatocytes
Source: PLoS One. 2014 Sep 8;9(9):e105930. doi: 10.1371/journal.pone.0105930 (PMC4157742; doi:10.1371/journal.pone.0105930)
Supplement: Table S1 — Summary of PHH Donors. (DOCX) [file pone.0105930.s002.docx]

| Donor Serial # | **Gender** | **Age** | **Diagnosis** | **RNA Induction** | **ChIP Enrichment** |
| --- | --- | --- | --- | --- | --- |
| 1st | Female | 58 | N/A | Good | N/A ^§^ |
| 2nd | Female | 85 | Metastatic colon cancer and chemotherapy | Good | N/A ^§^ |
| 1956 | Male | Pediatric | Donor, fatty | Fair | N/A ^§^ |
| 1958 * | Female | ~ 60 | 5-10% steatosis | Good | Good |
| 1959 * | Male | Mid age | Metastatic cancer, prior chemotherapy | Good | Good |
| 1962 * | Female | ~ 40 | Adenocancer metastic to liver | Good | Good |
| 1974 * | Male | 56 | Organ donor | Good | Good |
| 1983 | Male | 70 | Organ donor | Good | Fair |
| MSUD | N/A | N/A | Maple syrup urine disease | Good | Good |

**Table S1. Summary of PHH Donors**

* Selected PHH donors for ChIP-seq library preparation, which had enough chromatin yields to pool from both DMSO and GW4064 treatment. RNA samples from these four donors were also used and pooled for RNA-seq. Patient I.Ds, from 1956 to MSUD, was used by the tissue bank to record individual PHH. No personal identity information was obtained nor provided by the provider.

**^§^** N/A stands for not available. Due to limited quantity of cells, we didn’t detect valid Ct values for the positive controls (promoter regions of FXR target genes) from the ChIP-qPCR assay for these PHH donors.
