# Supplementary material for: Genome-Wide Binding and Transcriptome Analysis of Human Farnesoid X Receptor in Primary Human Hepatocytes
Source: PLoS One. 2014 Sep 8;9(9):e105930. doi: 10.1371/journal.pone.0105930 (PMC4157742; doi:10.1371/journal.pone.0105930)
Supplement: Table S2 — Primers Used for Quantitative PCR. (DOCX) [file pone.0105930.s003.docx]

**Table S2. Primers Used for Quantitative PCR**

| Primer Name | Forward Primer Sequence (5' - 3') | Reverse Primer Sequence (5' - 3') | Amplicon |
| --- | --- | --- | --- |
| RT Human 18S | GAGCGAAAGCATTTGCCAAG | GGCATCGTTTATGGTCGGAA | 101 |
| RT Human BSEP | AGTTGCTCATCGCTTGTCTACG | GCTTGATTTCCCTGGCTTTG | 153 |
| RT Human OST-β | GCAGCTGTGGTGGTCATTAT | TAGGCTGTTGTGATCCTTGG | 490 |
| RT Human FXR | CGCCTGACTGAATTACGGACA | TCACTGCACGTCCCAGATTTC | 114 |
| ChIP Human BSEP | TTCACAACCTTTTCCAACCTCGGTT | TGTCACTGAACTGTGCTTGGGCTG | 131 |
| ChIP Human OST-β | AATGAAAGCACTGGGCTACTGGTG | TCCAGGGTGACTGACCTCTTGAAT | 99 |
| ChIP Human IL-8 | ACTCAGGTTTGCCCTGAGGGGA | TGCCTTATGGAGTGCTCCGGTG | 136 |
| ChIP Human ACTBP11 | GCTTGGTGGCTGAAGAGTGA | ACCCCATGTAATCACGAGGC | 106 |
| ChIP Human AOC1 | GATTCTGTAGCGCCGAGTCA | ATCCTGTACCCTCTACCCCG | 293 |
| ChIP Human FABP3 | GGGCAGCCACCTTTATCCAA | TCTTCCCACCAAGCCTTAGC | 296 |
| ChIP Human HS6ST1 | CTCTGATGGCCCTCCTGTTG | AGCCATGGCCACTCATAACC | 250 |
| ChIP Human GFOD2 | TTTGGCAGGTTCTGGGACTC | GAGAATCTGTCCACTGCCCC | 120 |
| ChIP Human PNMT | ATCCGCATCCAGGGTTTGTT | AAGTCTCCTTGGGAGAGGCA | 295 |
| ChIP Human UROC1 | CTGGGGAGGACATTGCTCTG | AGATGCAGATCCCCACATCG | 188 |
