# Supplementary material for: Genome-Wide Binding and Transcriptome Analysis of Human Farnesoid X Receptor in Primary Human Hepatocytes
Source: PLoS One. 2014 Sep 8;9(9):e105930. doi: 10.1371/journal.pone.0105930 (PMC4157742; doi:10.1371/journal.pone.0105930)
Supplement: Table S3 — Comparison of Genes from Selected Categories in DAVID Anotation for ChIP-seq. (DOCX) [file pone.0105930.s004.docx]

**Table S3. Comparison of Genes from Selected Categories in DAVID Anotation for ChIP-seq***

| **A. KEGG Analysis** | | |
| --- | --- | --- |
| **Category** | **mLiver-GW** | **PHH-GW** |
| Retinol metabolism |  | *CYP3A4, CYP2B6, CYP2C9, CYP2C18, CYP3A43, DGAT1, CYP2A6, CYP2A7, ADH1B, ADH1A, CYP3A7, CYP1A1,UGT2B10* |
| Drug metabolism | *CYP2D9, CYP3A25, CYP2A12, FMO5, GSTM3, UGT1A7C, ADH4, GSTK1, FMO3, GSTZ1, UGT1A@, GSTA2,GSTA4, CYP3A13, CYP3A11, GSTT1, GSTT2, CYP2E1, UGT1A1, UGT1A10, AOX1, CYP2D26, CYP2C38, CYP2C39, MGST1, GSTA3* | *CYP3A4, CYP2B6, CYP2C9, CYP2C18, ADH1B, CYP3A43, CYP2A6, CYP2A7, CYP3A7,CYP2D6, ADH1A,UGT2B10, GSTA3* |
| Complement and coagulation cascades | *KNG1, MBL1, KNG2, HC, C3, C4B, CFB, SERPINA1E, PLG, PROC, SERPINF2,CFH, C2, CFD, FGG, FGA, FGB, F2, SERPINE1, SERPINC1, CPB2* | *F11, MBL2, SERPINA1, BDKRB2, FGG, FGA, FGB, F2, SERPINE1, SERPINC1, CPB2* |
| Metabolism of xenobiotics by cytochrome P450 | *GSTA2, GSTA3, GSTA4, CYP3A25, CYP3A13, CYP3A11, GSTT1, GSTT2, CYP2E1, UGT1A1, DHDH, UGT1A10, GSTM3, UGT1A7C, GSTK1, ADH4, GSTZ1, UGT1A@, CYP2C38, CYP2C39, MGST1* | *CYP3A43, CYP3A4, GSTA3, CYP3A7, CYP1A1, CYP2C18, CYP2C9, CYP2B6, ADH1B, ADH1A, UGT2B10* |
| PPAR signaling pathway | *ACOX2, PPARD,PPARA, APOA2, APOA5,APOC3,PCK1(HepG2), CPT1A, CPT1B, FABP5, APOA1, ACSL1, CYP7A1, ACAA1B, ACSL5, ANGPTL4, SCD1, ACADM, DBI, ADIPOQ, CYP27A1, UBC, CYP4A14, SLC27A2, SCP2, SLC27A4* | *PPARA, APOA2, APOA5,APOC3,CPT2, FABP1, ANGPTL4* |
| Steroid hormone biosynthesis | *CYP3A25, CYP3A13, HSD17B2, HSD17B1, CYP3A11, CYP21A1, UGT1A1, UGT1A10, CYP17A1, UGT1A7C, CYP7A1, SRD5A1, SULT1E1, UGT1A@* | *CYP3A43, CYP3A4, CYP3A7, HSD17B2, CYP1A1, UGT2B10* |
| Circadian rhythm | *NPAS2, CSNK1D, PER1, PER3, ARNTL, CRY1* | *NPAS2, BHLHE40, CRY1* |
| Linoleic acid metabolism | *CYP2J5, CYP3A25, CYP3A13, CYP3A11, PLA2G6, CYP2E1, CYP2C38, CYP2C39* | *CYP3A43, CYP3A4, CYP3A7, CYP2C18, CYP2C9, PLA2G6* |
| Glycerophospholipid metabolism | *CHPT1, GPD1, PEMT, PLA2G6, AGPAT2, AGPAT1, CRLS1, NAT6, PPAP2C, CHKB, LYPLA2, LCAT, PHOSPHO1,* | *CHPT1, GPD1, PEMT,PLA2G6, AGPAT2, AGPAT6* |
| **B. GO-BP Analysis** | | |
| **Category** | **mLiver-GW** | **PHH-GW** |
| organic ether metabolic process | *MOGAT2, G6PC, APOA5, APOC1, LIPC, APOC3, SLC37A4, SLC22A4, LIPE, PCK1, INSIG2,* | *CYP1A1, IL6ST, APOC1, APOC2, AGPAT6, DGAT1, APOE, G6PC, APOC3, APOA2, APOA4, APOA5, LIPC* |
| monocarboxylic acid transport | *CPT1B, PPARD, SLC6A6, ABCC3, SLC27A2, CROT, SLC10A1, SLC27A4* | *MIP, SLC16A5, PPARA, CPT2, PLIN2, AQP8, FABP1, BDKRB2, SLC10A1* |
| regulation of cellular ketone metabolic process | *PPARA, HNF4A, INSIG2, AGT, MLXIPL, GNMT, ADIPOQ, BRCA1* | *APOA4, PPARA, AGT, APOA5, APOC3, APOC1, APOC2, CPT2, FABP1* |
| lipid transport | *RBP4, OSBP, PPARD, LDLR, APOC1, APOC2, APOA4, APOA2, APOA1, APOE, LCAT, APOC4, APOC3, APOA5, ATP8B1, LBP, OSBPL5, CPT1B, ABCG8, NPC1, ABCG5, LIPC, SLC27A2, SCP2, CROT, SLC27A4* | *PPARA, LDLR, APOC1, APOC2, APOA4, APOA2, P2RX7, APOE, APOC4, APOA5, APOC3, LBP, LIPC, BDKRB2, CPT2, FABP1, GLTPD2,PLIN2* |
| triglyceride metabolic process | *MOGAT2, G6PC, INSIG2, APOA5, APOC3, SLC37A4, APOC1, SLC22A4, LIPC, LIPE, PCK1* | *APOA4, APOA2, G6PC, AGPAT6, DGAT1, APOE, IL6ST, APOA5, APOC3, APOC1, APOC2, LIPC* |
| glycerolipid metabolic process | *MOGAT2, ALDH5A1, CHKB, SLC37A4, APOC1, PTEN, CHPT1, PCK1, G6PC, INSIG2, PIGG, APOC3, APOA5, PEMT, SLC22A4, ETNK2, LIPC, IPMK, ALG12, FABP5, LIPE* | *GPD1, ALDH5A1, IL6ST, APOC1, APOC2, CHPT1, APOA4, APOA2, AGPAT6, G6PC, DGAT1, APOE, APOA5, APOC3, PEMT, PLA2G6, LIPC, AGPAT2, IP6K3* |
| chemical homeostasis | *SLC9A8, PPARD, GCLC, FTL1, LDLR, ATOX1, SLC37A4, NR3C2, AQP4, TTC7, TRF, ASGR2, APOA2, GCKR, SLC24A3, APOE, GRIN2C, PXMP3, APOA5, MT2, LGI4, MT1, EIF2B4, SCO1, PRKCA, IBTK, SLC8A1, STIM2, MLXIPL, BAD, CSRP3, ADIPOQ, PARK7, USF2, QK, ATXN1, ABCG8, NPC1, G6PC, TSC1, LYST, VEGFA, LIPC, NR5A2, CLN6* | *FXYD1, GNA13, LDLR, IL6ST, ATP5B, OXT, APOC2, BDKRB2, TCF7L2, APOA4, GCKR, APOA2, NUBP1, SAA1, APOE, AGT, APOC4, APOA5, SERPINE1, APOC3, TGM2, QKI, PPP3CA, IBTK, P2RX7, G6PC, CCL14, F2, MT2A, CP, LIPC* |
| steroid metabolic process | *SC5D, OSBP, LDLR, MVD, HSD17B2, HSD17B1, SLC37A4, STAT5B, APOC1, RDH9, ACBD3, APOA2, APOA1, INSIG2, APOE, SAA1, SERPINA6, CYP7A1, LCAT, PXMP3, SULT1A1, APOC3, ATP8B1, SRD5A1, SULT1E1, DHCR24, OSBPL5, CYP21A1, AMACR, ESR1, RDH1, AFP, NPC1, G6PC, CYP17A1, PON1, LIPC, NR5A2, CLN8, LIPE, CLN6* | *CYP3A4, CYP1A1, HSD17B2, MVD, LDLR, APOC1, NR0B2, SREBF2, APOA4, APOA2, G6PC, APOE, SULT1A1, APOC3, INSIG1, LIPC* |
| fatty acid metabolic process | *PTGES3, ACOX2, PRKAG3, HACL1, PPARA, PPARD, SC5D, PRKAG2, STAT5B, ACOT5, ACOT4, ACOT3, PECR, APOA2, ACSL1, ELOVL5, FASN, ACOT12, ELOVL6, ACAA1B, ACSL5, SCD1, CPT1B, ACADM, ALDH5A1, EPHX2, ADIPOR2, LYPLA2, PHYH, ADIPOQ, CPT1A, BRCA1, QK, PTGDS, MAPK14, LIPC, AACS, SLC27A2, CROT, SLC27A4, DEGS1* | *PPARA, AGPAT6, CPT2, ECH1, ALDH5A1, ELOVL2, FASN, QKI, CYP4F3, LIPC, ACOT4* |
| oxidation reduction | *CYP2D9, ACOX2, CYP2J5, STEAP3, LDHA, SC5D, ALDH1L1, PRDX5, PDHB, GPX2, RDH9, PECR, CYP7A1, CPOX, SRD5A1, DHTKD1, DUS1L, SARDH, GFOD1, DHCR24, HPD, SQRDL, ACADM, CYP3A13, ALDH5A1, CYP3A11, CYCS, DECR2, QDPR, CYP26A1, RDH1, CYP2E1, GRHPR, CDO1, POR, DHDH, CYP27A1, H6PD, SLC37A2, CYP2D26, TXNRD2, DEGS1, CYP2U1, XDH, CYP3A25, NDUFB6, HSD17B2, HSD17B1, HSD17B13, AASS, MOSC2, EGLN2, KMO, PAH, ALDH3A2, PIPOX, CYP2A12, FMO5, ADH4, FMO3, HAAO, FASN, BDH1, BCKDHA, SCD1, CYP2G1, GPD1, CHDH, CYP21A1, HGD, PHYH, AKR1B7, CYP17A1, SLC25A13, LEPRE1, UOX, NDUFV1, CYP4F15, AOX1, PRODH2, CP, CYP2C38, CYP4A14, ACAD10, ALKBH2, CYP2C39* | *STEAP3, CYP3A4, STEAP4, CYP3A7, HSD17B2, CYP2B6, CYP2C18, CYP2D7P1, CYP2D6, ADH1B, ADH1A, CYP3A43, PLOD2, PLOD3, FASN, LOXL4, SARDH, GFOD2, DUS3L, GPD1, PAOX, CYP1A1, CYP2C9, PYROXD2, ALDH5A1, CYB5A, IYD, SLC25A13, RRM2, CYP2A6, CYP4F3, CYP2A7, CP, KDM6B, AOC3* |
| response to wounding | *PPARA, JUB, PPARD, TRPV1, CRP, TLR3, SAA2, GRIN2C, SAA1, TICAM1, PROZ, CFH, LBP, CFD, KNG1, C4B, SAA3, SAA4, PROC, SERPINF2, LYST, F2, CTSB, LCP1, MBL1, RTN4RL1, C3, CXCL2, NINJ1, ABHD2, ITGB2, TRF, AHSG, FGG, IL17B, FGA, FGB, MAP3K1, SERPINC1, C2, PAPSS2, B4GALT1, LIPA, CFB, HC, SAAL1, EPHX2, PLG, ORM1, C1RL, HBEGF, ORM2, AI182371* | *GNA13, MBL2, PPARA, NMI, NDST1, TGFB3, ITGB3, BDKRB2, APOA2, FGG, FGA, FGB, SAA1, SERPINE1, APOA5, SERPINC1, SERPINA1, LBP, F11, CEBPB, CYP1A1, CCNB1, ORM1, P2RX7, SDC1, TSC2, F2, KDM6B, ORM2, AOC3* |
| cellular amino acid derivative metabolic process | *AHCY, GCLC, CHKB, STAT5B, AGMAT, AFMID, OAZ1, CSAD, GSTK1, SLC22A4, PEMT, ETNK2, GNMT, CHDH, P4HB, ACADM, ALDH5A1, NR4A2, GSTT1, GSTT2, GSTT3, CDO1, TPMT, CHPT1, GAMT, LIPC, PTMS, FABP5, MGST1* | *HAGH, APOA4, APOA2, AGPAT6, CKM, CYP1A1, PNMT, ALDH5A1, SULT1A1, PEMT, LIPC, CHPT1* |
| homeostatic process | *SLC9A8, PPARD, LDLR, ATOX1, TRPV1, STAT5B, PRDX5, AQP4, TENC1, GPX2, ASGR2, APOA2, DNAJC16, SLC24A3, APOE, GRIN2C, PXMP3, APOA5, MT2, MT1, LGI4, FAS, EIF2B4, PRKCA, IBTK, SLC12A7, MLXIPL, STIM2, QK, NPC1, G6PC, LYST, VEGFA, PDGFRB, TXNRD2, CLN6, XDH, GCLC, FTL1, STK11, CSF1, SLC37A4, NR3C2, EGLN2, SFXN1, TTC7, HSPA1A, TRF, ZC3H8, GCKR, FH1, EPO, SCO1, P4HB, SLC8A1, LIPA, SMG6, BAD, CSRP3, ADIPOQ, PLG, USF2, PARK7, ATXN1, ABCG8, TSC1, ID2, RHOT1, NR5A2, LIPC* | *FXYD1, GNA13, SIVA1, LDLR, IL6ST, ATP5B, OXT, APOC2, BDKRB2, TCF7L2, APOA4, ADRB3, GCKR, APOA2, NUBP1, APOE, SAA1, APOC4, AGT, APOA5, SERPINE1, APOC3, TGM2, QKI, PPP3CA, IBTK, SLC12A7, FOXP3, TXNDC11, P2RX7, G6PC, CCL14, F2, MT2A, CP, LIPC, CLCN6* |
| cholesterol metabolic process | *MVD, LDLR, APOC1, APOA2, APOA1, INSIG2, APOE, SAA1, LCAT, CYP7A1, APOC3, PON1, LIPC, CLN8, LIPE, CLN6, DHCR24* | *APOA4, APOA2, LDLR, MVD, APOE, APOC3, INSIG1, APOC1, NR0B2, LIPC, SREBF2* |
| acute-phase response | *TRPV1, SAAL1, CRP, SAA3, SAA4, TRF, AHSG, ORM1, SAA2, SERPINF2, SAA1, F2, LBP, ORM2* | *ORM1, MBL2, CEBPB, SAA1, F2, TSC2, SERPINA1, LBP, ORM2* |
| acute inflammatory response | *MBL1, C3, TRPV1, CRP, TRF, AHSG, SAA2, SAA1, CFH, C2, LBP, CFD, B4GALT1, HC, CFB, C4B, SAAL1, EPHX2, SAA3, SAA4, ORM1, SERPINF2, F2, C1RL, ORM2, AI182371* | *ORM1, MBL2, APOA2, CEBPB, SAA1, F2, TSC2, SERPINA1, LBP, ORM2* |
| coenzyme metabolic process | *ALDH1L1, GCLC, KMO, PDSS1, ACOT5, PIPOX, ACOT4, GCH1, ACOT3, GSTK1, ACOT12, HAAO, FH1, SUCLA2, NAPRT1, CES3, ALDH5A1, PDK4, GSTT1, GSTT2, ACLY, GSTT3, DLAT, HNF4A, PANK1, H6PD, FPGS, FLAD1, SCP2, MGST1* | *HAGH, GPD1, MTHFS, AGPAT6, PANK3, ALDH5A1, FTCD, ACLY, ACOT4* |
| organic acid catabolic process | *ACOX2, HACL1, BCKDK, PPARD, AHCY, AASS, PAH, FAH, AFMID, CSAD, GSTZ1, SARDH, HPD, BCKDHA, ACADM, HAL, FTCD, HGD, CDO1, TAT, ADIPOQ, PHYH, AMDHD1, PRODH2, UROC1, SLC27A2, SLC27A4* |  |
| glucose metabolic process | *PTGES3, PRKAG3, RBP4, PPARA, LDHA, SLC37A4, CAR5A, PDHB, PPP1R3B, NISCH, GYS2, GNMT, DHTKD1, ENO1, GPD1, ALDH5A1, PDK4, BAD, DLAT, PPP1CC, ADIPOQ, CPT1A, PCK1, PGM2, PCX, G6PC, GBE1, H6PD, PYGL, SDS, MAPK14, FABP5* |  |
| cellular amino acid catabolic process | *BCKDHA, BCKDK, AHCY, HAL, FTCD, HGD, AASS, PAH, CDO1, TAT, FAH, AFMID, AMDHD1, CSAD, PRODH2, GSTZ1, UROC1, SARDH, HPD* |  |
| hexose metabolic process | *PTGES3, PRKAG3, RBP4, PPARA, LDHA, GNPDA1, SLC37A4, CAR5A, PDHB, PPP1R3B, NISCH, GYS2, GALE, GNMT, DHTKD1, ENO1, B4GALT1, GPD1, ALDH5A1, PDK4, BAD, DLAT, PPP1CC, ADIPOQ, CPT1A, PCK1, PGM2, PCX, G6PC, GBE1, H6PD, PYGL, SDS, MAPK14, FABP5* |  |
| monosaccharide metabolic process | *PTGES3, PRKAG3, RBP4, PPARA, LDHA, GNPDA1, SLC37A4, CAR5A, PDHB, NISCH, PPP1R3B, UGT1A7C, GYS2, GALE, GNMT, UGT1A@, DHTKD1, ENO1, B4GALT1, GPD1, ALDH5A1, PDK4, BAD, DLAT, PPP1CC, ADIPOQ, UGT1A1, CPT1A, PCK1, PGM2, UGT1A10, PCX, G6PC, GBE1, H6PD, PYGL, SDS, MAPK14, FABP5* |  |
| L-phenylalanine catabolic process | *HGD, GSTZ1, PAH, TAT, HPD, FAH* |  |
| pyruvate metabolic process | *RBP4, GPD1, PCX, G6PC, SDS, PDK4, CAR5A, DLAT, AGXT, PCK1* |  |

* Detailed list of genes retrieved from DAVID analyses for **Table 2**. For GO-BP analysis, the same categories are listed here as in **Table 2**. For KEGG analysis, categories with FDR > 0.1 are also included. The orders of the categories listed here are the same as in **Table 2,** based on the FDR values from PHH-GW dataset. Please notice that all mouse genes were kept in capitalized state as retrieved directly from DAVID analyses.
